# Tissue Kallikrein Alleviates Cerebral Ischemia-Reperfusion Injury by Activating the B2R-ERK1/2-CREB-Bcl-2 Signaling Pathway in Diabetic Rats

**DOI:** 10.1155/2016/1843201

**Published:** 2016-06-30

**Authors:** Ruifeng Shi, Kunxiong Yuan, Bin Hu, Hongfei Sang, Lizhi Zhou, Yi Xie, Lili Xu, Qinqin Cao, Xin Chen, Lingling Zhao, Yunyun Xiong, Gelin Xu, Xinfeng Liu, Ling Liu, Renliang Zhang

**Affiliations:** ^1^Department of Neurology, Jinling Hospital, Medical School of Nanjing University, Nanjing, Jiangsu 210002, China; ^2^Department of Neurology, Maanshan Municipal People's Hospital, Maanshan, Anhui 243000, China; ^3^Department of Neurology, Jinling Hospital, Southern Medical University, Nanjing, Jiangsu 210002, China; ^4^Department of Orthopedics, Maanshan Municipal People's Hospital, Maanshan, Anhui 243000, China; ^5^State Key Laboratory of Organ Failure Research, National Clinical Research Center for Kidney Disease, Department of Biostatistics, School of Public Health and Tropical Medicine, Southern Medical University, 510515 Guangzhou, China

## Abstract

Diabetes mellitus (DM) substantially increases the risk of ischemic stroke and reduces the tolerance to ischemic insults. Tissue kallikrein (TK) has been demonstrated to protect neurons from ischemia/reperfusion (I/R) injury in orthoglycemic model by activating the bradykinin B2 receptor (B2R). Considering the differential effects of B2R or bradykinin B1 receptor (B1R) on cardioprotection and neuroprotection in I/R with or without diabetes, this study was designed to investigate the role of TK during cerebral I/R injury in streptozotocin-induced diabetic rats. Intravenous injection of TK inhibited apoptosis in neurons, alleviated edema and inflammatory reactions after focal cerebral I/R, significantly reduced the infarct volume, and improved functional recovery. These beneficial effects were accompanied by activation of the extracellular signal-regulated kinase 1/2 (ERK1/2), cAMP response element-binding (CREB), and Bcl-2 signal proteins. Inhibition of the B2R or ERK1/2 pathway abated the effects of TK, whereas an antagonist of B1R enhanced the effects. These findings reveal that the neuroprotective effect of TK against cerebral I/R injury in streptozotocin-induced diabetic rats mainly involves the enhancement of B2R and ERK1/2-CREB-Bcl-2 signaling pathway activity.

## 1. Introduction

Ischemic stroke is the third leading cause of disability-adjusted life years worldwide [1]. Diabetes mellitus (DM) is one definitive risk factor of stroke and has been diagnosed in more than 30% of ischemic stroke patients [2]. Indeed, stroke survivors with diabetes have a higher risk of recurrence and a poorer prognosis compared with nondiabetics [3–6]. Although intensive glycemic control decreases the infarct volume and improves functional recovery after stroke [7], early recanalization is the only evidence-based effective therapy for improving the clinical outcomes of acute ischemic stroke [8]. Ischemia/reperfusion (I/R) injury, as one serious complication of restoring blood flow to the ischemic cerebrum, may offset the benefits of recanalization, especially in diabetes cases [9]. DM is known to exacerbate ischemic injury and to impede functional recovery by elevating inflammatory cytokines, promoting leukocyte infiltration, and accelerating the subsequent apoptotic cascade and neuronal death [5, 10–12]. Moreover, the cerebrum of diabetics is resistant to the neuroprotective effects of ischemic pre- or postconditioning or the administration of neuropharmacological agents [13]. Thus, further evaluation of the effects of DM is necessary to develop novel protective therapies for cerebral ischemia.

Kinins derived from kininogen produced by tissue kallikrein (TK) exert a broad spectrum of cellular functions via activation of the bradykinin B1 or B2 receptor (B1R or B2R, resp.) [14]. The physiological and pathological effects of TK have been shown to involve several cellular signaling pathways. Our previous studies have shown that TK could alleviate glutamate-induced neurotoxicity and protect cortical neurons against I/R and hypoxia/reoxygenation injury via the extracellular signal-regulated kinase 1/2 (ERK1/2) pathway [15–17]. In nondiabetic animals, TK was shown to improve neurofunctioning after ischemic stroke by inhibiting the NF-*κ*B signal pathway, activating the ERK1/2 pathway [18, 19], while in diabetic rats, TK attenuates insulin resistance and diabetic nephropathy via activation of phosphatidylinositol 3-kinase/protein kinase B [20]. Furthermore, increased TK plasma concentrations might exert greater cardioprotection in type 2 DM patients than that in non-DM patients [21]. Recently, we found a detrimental role of B1R and a beneficial effect of B2R in diabetic cerebral ischemia [22]. The present study aimed to assess whether TK could ameliorate cerebral I/R injury in streptozotocin-induced diabetic rats and investigate the roles of the ERK1/2 signaling pathway during acute ischemia and early reperfusion.

## 2. Materials and Methods

### 2.1. Establishment of the Diabetes Model

Male Sprague-Dawley rats (100–110 g) were purchased from the Animal Center of Jinling Hospital. All procedures were performed in accordance with the National Institutes of Health Guide for the Care and Use of Laboratory Animals (NIH Publications Number 80–23, revised 1996) and under the approval of the Institutional Animal Care and Use Committee of Nanjing University. All animals were placed on a 12/12-hour light/dark schedule and housed in a temperature- (22 ± 2°C) and humidity-controlled (55 ± 5%) room with free access to food (high-fat diet) and water. Diabetes was induced with streptozotocin (Sigma-Aldrich, St. Louis, MO, USA) at a dose of 35 mg/kg after three weeks of high-fat diet feeding, as previously described [23]. Two weeks after the injection of streptozotocin, rats with fasting blood glucose concentrations ≥ 16.67 mmol/L (300 mg/dL) in three separate measures were considered diabetic.

### 2.2. Focal Cerebral Ischemia

Middle cerebral artery occlusion (MCAO) and reperfusion models in rats were established as previously described [24]. After 90 min of MCAO, the filament was withdrawn to restore cerebral blood flow (CBF) through the left MCA. CBF was monitored continuously with a PeriFlux Laser Doppler System 5000 (Perimed AB, Sweden) throughout the operation to confirm proper occlusion and reperfusion. After full recovery from anesthesia, rats were tested with the Longa score. The scores of 1 (failure to extend left forepaw fully), 2 (circling to the left), and 3 (falling to the left) represent mild focal neurologic deficit, moderate focal neurologic deficit, and severe focal deficit, respectively. The animal with a score of 4 could not walk spontaneously and experienced coma and confusion. The rats that attained a score of 0 were excluded from the study, which indicated noneurologic deficit. Sham-operated rats underwent the same operation without the insertion of the filament.

### 2.3. Experimental Groups

**Figure 1:**
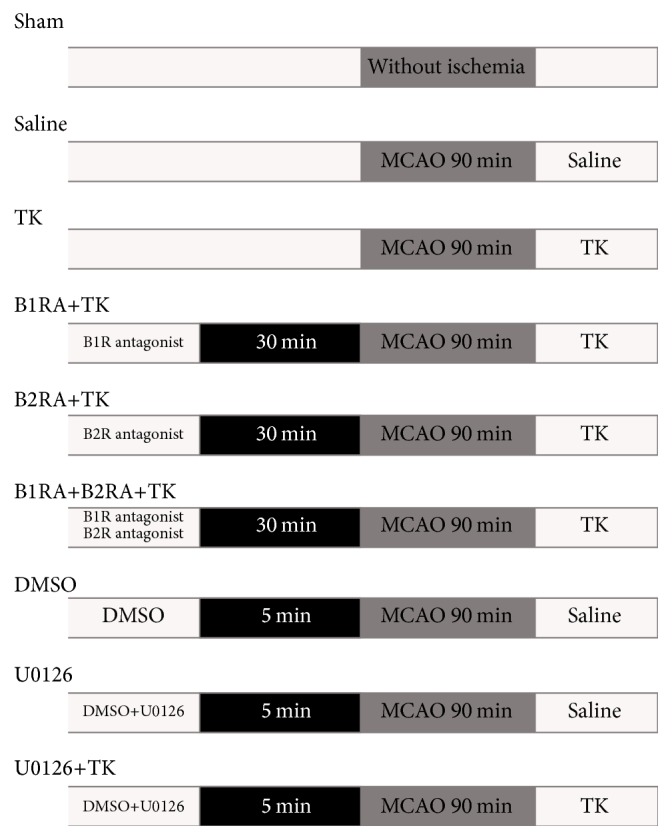
The experimental grouping and protocol.

Animals were randomized into nine groups: sham operation, saline, TK, TK+B1RA (B1R antagonist), TK+B2RA (B2R antagonist), TK+B1RA+B2RA, DMSO, U0126 (ERK1/2 inhibitor), and TK+U0126. Either the B2R antagonist bradyzide (1 nmol/kg, Sigma-Aldrich) or B1R antagonist Lys-(des-Arg9-Leu8)-bradykinin (300 nmol/kg, Sigma-Aldrich) was injected 30 min before the operation, whereas TK (1.6 × 10^−2 ^PNAU/kg, Techpool Bio-Pharma Co. Ltd., Canton, China) [25] or saline (2 mL/kg) was given intravenously immediately after reperfusion. U0126 (9903, Cell Signaling Technology, Danvers, USA), an inhibitor of the ERK1/2 signaling pathway, was dissolved in dimethyl sulfoxide (DMSO, Sigma-Aldrich, St. Louis, MO, USA) at 10 mmol/L and was subsequently diluted with saline for use. U0126 (400 *μ*g/kg) or an equal amount of DMSO was administered intravenously 5 min before the operation [26]. The experimental grouping and protocol are described in the flow diagram (Figure 1). Previous studies demonstrated that the expression of B1R and B2R in cerebral ischemia/reperfusion injury peaks at 24 h after reperfusion in diabetes [22]. Therefore, 24 h after reperfusion was chosen as the time point for research in this study.

### 2.4. Assessment of Neurological Deficits

Neurological deficits were evaluated by an investigator blinded to the groupings at 24 h after reperfusion using the neurological severity scores (NSS) [27], which included motor test, sensory test, beam balance test, and examinations of reflexes and abnormal movements.

### 2.5. Estimation of Brain Edema

Rats were sacrificed 24 h after reperfusion. The hemispheres were weighed to obtain the wet weight (WW), and their dry weights (DW) were measured after desiccation at 105°C for 24 h. The brain moisture content (%) was calculated as previously described [22].

### 2.6. Evaluation of Infarct Volume

Brains were removed 24 h after reperfusion and cut into six 2 mm thick coronal slices. The infarct volume was evaluated by 2,3,5-triphenyltetrazolium chloride (TTC, Sigma-Aldrich, St. Louis, MO, USA) staining and analyzed according to our previous protocol [22].

### 2.7. Histopathological Study

For histopathological study, rats were perfused with 200 mL of 0.9% saline and 200 mL 4% paraformaldehyde (PFA, pH 7.4) successively through the left ventricle 24 h after reperfusion. Then, the cerebral hemisphere was collected and fixed with 4% PFA. After gradient elution with sucrose, the brain was quickly frozen and cut into 14 *μ*m sections.

#### 2.7.1. Fluoro-Jade C Staining (FJC)

Slides were immersed in a basic alcohol solution consisting of 1% NaOH in 80% ethanol for 5 min and were then rinsed in 70% ethanol for 2 min, followed by incubation in 0.06% potassium permanganate solution for 10 min. The sections were then immersed in 0.0001% working solution of FJC (dissolved in 0.1% acetic acid vehicle) for 15 min. After washing and drying, the sections were cleaned in xylene and cover-slipped with DPX (Sigma-Aldrich, St. Louis, MO, USA) nonfluorescent mounting media. The data are expressed as the number of FJC-positive cells counted per section relative to the sham group.

#### 2.7.2. TUNEL Staining

The terminal deoxynucleotidyl transferase-mediated dUTP nick-end labeling (TUNEL) assay (Roche, Indianapolis, IN, USA) was used to assess apoptosis in neurons. Slides were postfixed with 4% PFA for 20 min and permeabilized with 0.1% Triton X-100 for 2 min on ice. After incubating in 15% glacial acetic acid for 2 min, the sections were treated with TUNEL mixture (enzyme solution : label solution = 1 : 9) for 60 min at 37°C in a dark humidified atmosphere, followed by converter-AP for 30 min. After washing with PBS three times for 5 min each, the specimens were incubated with BICP/NBT (Alkaline Phosphatase Color Development Kit, Beyotime, Shanghai, China) for 30 min and mounted under glass coverslips with CC/MOUNT. The data were expressed and analyzed in the same manner as for FJC staining.

#### 2.7.3. Immunohistochemical Examination

Slides were postfixed in 4% PFA for 15 min followed by 0.3% H_2_O_2_ for 30 min. After permeabilization with 0.1% Triton for 10 min, the sections were incubated with 5% albumin bovine (Generay biotech, Shanghai, China) for 1 h. Then, the sections were incubated overnight with the following primary antibodies: anti-ionized calcium-binding adapter molecule 1 (Iba1) (1 : 1,000, Wako, 019-19741, Osaka, Japan), anti-cleaved caspase-3 (1 : 100, Cell Signaling Technology, Danvers, USA), or anti-myeloperoxidase (MPO) (1 : 200, Abcam, 65871 Cambridge, England). The slides were washed and then incubated with biotinylated anti-rabbit IgG antibody (Jackson, 711065152, West Grove, PA, USA) (1 : 200) for 2 h at room temperature. After incubation with ABC solution (Vector labs, California, USA), the slides were stained with the DAB Peroxidase Substrate Kit (Vector labs, California, USA), followed by PBS washing once the desired color was achieved. Sections were cleaned in xylene and cover-slipped with neutral balsam. The data were expressed and analyzed in the same manner as for FJC staining.

### 2.8. Real-Time Quantitative Reverse Transcription Polymerase Chain Reaction

Total RNA was extracted from ischemic boundary region tissue at 24 h after reperfusion using the Trizol reagent (Sigma-Aldrich, St. Louis, MO, USA) and then reverse-transcribed into cDNA using a RevertAid First Strand cDNA Synthesis kit (Thermo Scientific, Waltham, MA, USA) following the manufacturer's recommendations. Quantitative real-time PCR was performed using a real-time PCR system (Agilent Technologies, Santa Clara, CA, USA) with a fluorescent dye (CW0956 UltraSYBR Mixture). Glyceraldehyde-3-phosphate dehydrogenase (GAPDH) was used as an endogenous reference gene. The normalized messenger RNA levels were previously described in detail [28]. Data are expressed as the ratio of the level of IL-1*β* or TNF-*α* to that of GAPDH in the treated groups relative to the sham group. The primer sequences for the genes were as follows: IL-1*β*: sense: 5′-AGACTTCACAGAGGATACCACCCAC-3′, antisense: 5′-CAATCAGAATTGCCATTGCACAA-3′; TNF-*α*: sense: 5′-AGCAAACCACCAAGCGGAGG-3′, antisense: 5′-CAGCCTTGTCCCTTGAAGAGAAC-3′; and GAPDH: sense: 5′-GCAAGTTCAACGGCACAG-3′, antisense: 5′-GCCAGTAGACTCCACGACAT-3′.

### 2.9. Western Blotting

Samples for western blotting were collected from brain infarction boundary regions. After extraction and quantification, equal amounts of protein were separated by sodium dodecyl sulfate-PAGE and then transferred to an immobilon-polyvinylidene fluoride membrane. The membranes were blocked using 5% milk for 2 h at room temperature and probed with the primary antibody overnight at 4°C. These primary antibodies included rabbit anti-phospho-ERK1/2 (1 : 5,000), rabbit anti-total-ERK1/2 (1 : 5,000), rabbit anti-phospho-cAMP response element-binding protein (CREB) (1 : 1,000), rabbit anti-total-CREB (1 : 1,000), rabbit anti-Bcl-2 (1 : 200), rabbit anti-Bax (1 : 400), rabbit anti-cleaved caspase-3 (1 : 1000), and rabbit anti-*β*-actin (1 : 5,000). The secondary antibody was anti-rabbit HRP-conjugated antibody (1 : 4,000). All primary antibodies were purchased from Cell Signaling Technology (Danvers, USA), with the exception of the Bcl-2 antibody purchased from Santa Cruz (Dallas, USA). Immunoblots were developed with the Immobilon ECL method (Millipore, Billerica, Massachusetts, USA). Gray bands were converted to density values using Image J software for quantification analysis.

### 2.10. Data Analysis and Statistics

All data except for the NSS values are expressed as mean ± SD. The neurologic tests scores were analyzed using the Kruskal-Wallis test followed by the Mann-Whitney *U* test with Bonferroni corrections. Differences between groups were compared using one-way analysis of variance with the Welch correction for unequal variances. Post hoc tests, such as Bonferroni or Dunnett's T3 multiple comparison tests, were used according to whether unequal variances were present. A two-sided *P* value of *P* < 0.05 was defined as significant.

## 3. Results

### 3.1. Modifications of Neurological Deficits

**Figure 2:**
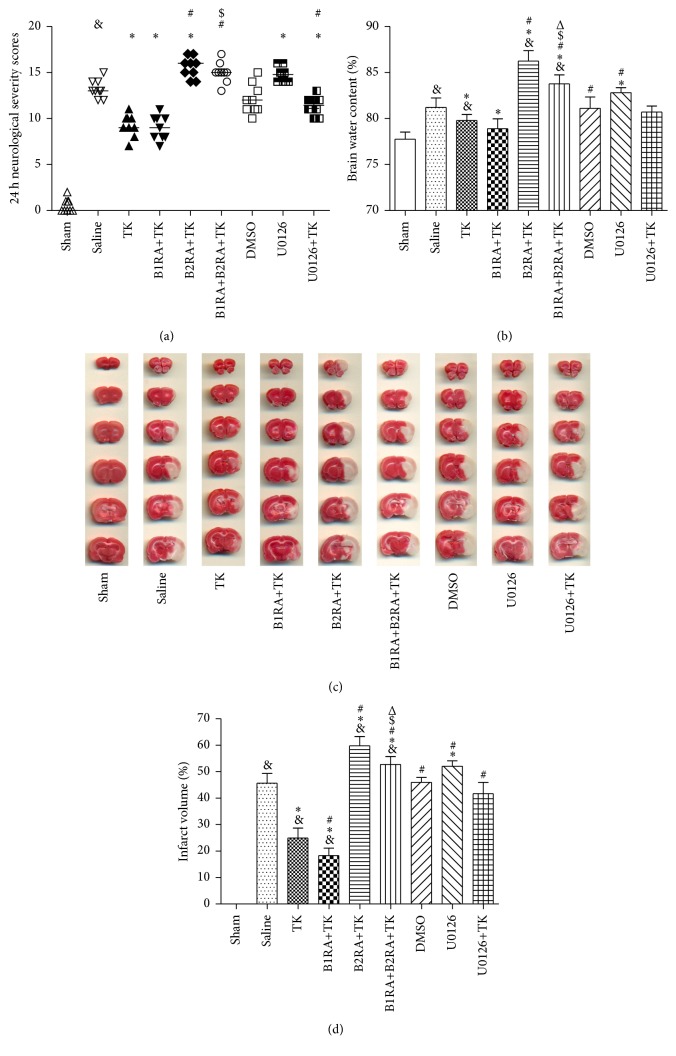
(a) NSS values recorded for each animal at 24 h after MCAO. *n* = 9 rats/group. (b) Brain edema recorded in each animal at 24 h after MCAO. Data are expressed as mean ± SD; *n* = 9 rats/group. (c) 2,3,5-Triphenyltetrazolium chloride staining recorded in nine groups at 24 h after MCAO; (d) the infarct volume percent at 24 h after MCAO. Data are expressed as mean ± SD; *n* = 7 rats/group (^&^ *P* < 0.05 versus sham group; ^*∗*^ *P* < 0.05 versus saline group; ^#^ *P* < 0.05 versus TK group; ^$^ *P* < 0.05 versus B1RA+TK group; ^Δ^ *P* < 0.05 versus B2RA+TK group).

At 24 h after MCAO, neurologic function improved in the TK group compared with the saline group (*P* < 0.05, Figure 2(a)). Compared with the TK group, the B1RA+B2RA+TK and B2RA+TK groups showed higher neurological deficit scores (*P* < 0.05), whereas the B1RA+TK group showed a comparable score (*P* > 0.05). Furthermore, neurological function in the B1RA+B2RA+TK group was worse than that of the B1RA+TK group (*P* < 0.05) but similar to that of the B2RA+TK group (*P* > 0.05). Treatment with the ERK1/2 inhibitor U0126 significantly aggravated neurological functioning (*P* < 0.05) and compromised the beneficial effect of TK (TK versus U0126+TK, *P* < 0.05).

### 3.2. Variation of Brain Edema

Brain edema at 24 h after reperfusion was significantly alleviated in TK group compared to saline group (*P* < 0.05, Figure 2(b)). B1R antagonist pretreatment had no interference on TK. However, pretreatment with the B2R antagonist hindered the efficacy of TK (*P* < 0.05). Brain edema in the B1RA+B2RA+TK group was more severe than that in the TK and B1RA+TK groups but less than that in the B2RA+TK group (*P* < 0.05). Administration of U0126 alone clearly increased the brain water content compared to rats in the saline group (*P* < 0.05). However, the brain water content in the U0126+TK group was not different from that in the TK group.

### 3.3. Change in Cerebral Infarct Volume

TK treatment remarkably reduced the infarct volume compared to saline at 24 h after reperfusion (*P* < 0.05, Figures 2(c) and 2(d)). Administration of B1RA+TK could further reduce the infarct volume of TK-treated animals, whereas B2RA+TK or B1RA+B2RA+TK treatment abolished the beneficial effect observed with the TK intervention (*P* < 0.05). Additionally, the infarct volume in the B1RA+B2RA+TK group was lower than that in the B2RA+TK group but higher than that in the B1RA+TK group (*P* < 0.05). Treatment with the ERK1/2 inhibitor U0126 increased the infarct volume in the saline group (*P* < 0.05). Furthermore, the infarct volume with U0126+TK treatment was higher than that with TK treatment alone (*P* < 0.05).

### 3.4. Expression of Iba1, MPO, and Proinflammatory Cytokines

**Figure 3:**
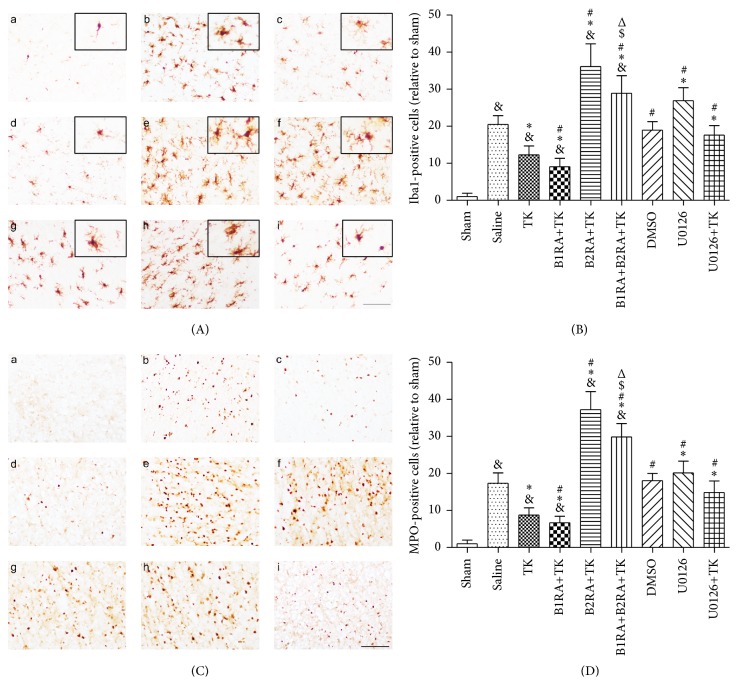
(A) Iba1 staining was performed 24 h after MCAO to observe microglia. (B) The number of Iba1-positive cells was counted at 24 h after MCAO. (C) MPO staining was performed 24 h after MCAO to observe neutrophils. (D) The number of MPO-positive cells was counted 24 h after MCAO. (a) Sham; (b) saline; (c) TK; (d) B1RA+TK; (e) B2RA+TK; (f) B1RA+B2RA+TK; (g) DMSO; (h) U0126; (i) U0126+TK. Scale bar = 100 mm. Data are expressed as mean ± SD; *n* = 3 rats/group. Six representative microscopic fields were analyzed for each rat (^&^ *P* < 0.05 versus sham group; ^*∗*^ *P* < 0.05 versus saline group; ^#^ *P* < 0.05 versus TK group; ^$^ *P* < 0.05 versus B1RA+TK group; ^Δ^ *P* < 0.05 versus B2RA+TK group).

Iba1 is a protein biomarker expressed in microglia that is upregulated during inflammatory responses after stroke in the infarction border zone. MCAO followed by 24 h of reperfusion induced a 20.46 ± 2.40-fold increase in Iba1-positive cells. Compared to saline, TK intervention significantly suppressed the activation of Iba1 (*P* < 0.05, Figures 3(A) and 3(B)). Furthermore, the efficacy of TK was enhanced by pretreatment with the B1R antagonist but was abolished by the B2R antagonist (*P* < 0.05). The number of Iba1-positive cells in the B1RA+B2RA+TK group was higher than that in the TK and B1RA+TK groups but lower than that in the B2RA+TK group (*P* < 0.05). In addition, U0126 treatment led to an increase in microglial activation (U0126 versus saline, *P* < 0.05), while pretreatment with U0126 abated the effect of TK (U0126+TK versus TK, *P* < 0.05). Neutrophil infiltration into the ischemic regions was investigated by immunohistochemical staining for MPO (Figures 3(C) and 3(D)), and the change in MPO expression was similar to that observed for Iba1.

**Figure 4:**
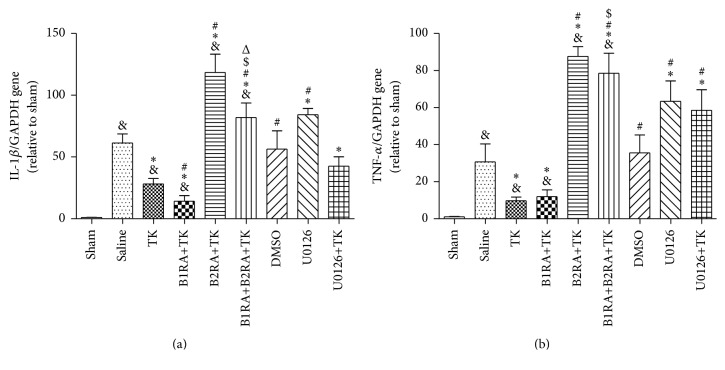
(a) The levels of IL-1*β* 24 h after MCAO. (b) The levels of TNF-*α* 24 h after MCAO. Values are expressed as mean ± SD; *n* = 6 rats/group (^&^ *P* < 0.05 versus sham group; ^*∗*^ *P* < 0.05 versus saline group; ^#^ *P* < 0.05 versus TK group; ^$^ *P* < 0.05 versus B1RA+TK group; ^Δ^ *P* < 0.05 versus B2RA+TK group).

Proinflammatory cytokines are expressed in the ischemic core in the early stage of the brain ischemic model. Reperfusion for 24 h after MCAO (saline group) led to a 61.27 ± 7.46-fold increase in IL-1*β* and a 30.71 ± 9.64-fold increase in TNF-*α* compared with the sham group (Figures 4(a) and 4(b)). Treatment with TK ameliorated this upregulation compared with the saline group (*P* < 0.05). Compared to TK-treated animals, B1R antagonism prior to MCAO attenuated IL-1*β* expression, whereas B2R antagonism elevated the levels of IL-1*β* and TNF-*α* (*P* < 0.05). Simultaneous antagonism of B1R and B2R before TK treatment induced much higher levels of IL-1*β* and TNF-*α* than TK intervention alone (*P* < 0.05). Furthermore, the relative expression of IL-1*β* and TNF-*α* in the B1RA+TK group was lower than that in the B1RA+B2RA+TK group (*P* < 0.05), whereas the level of IL-1*β* in the B1RA+B2RA+TK group was lower than that in the B2RA+TK group (*P* < 0.05). After injection of the ERK1/2 inhibitor U0126, the relative expression of TNF-*α* and IL-1*β* was dramatically increased compared to the saline group (*P* < 0.05). Additionally, pretreatment with U0126 elevated the level of TNF-*α* in rats that received TK treatment (TK versus U0126+TK, *P* < 0.05).

### 3.5. Neuronal Degeneration and Apoptosis

**Figure 5:**
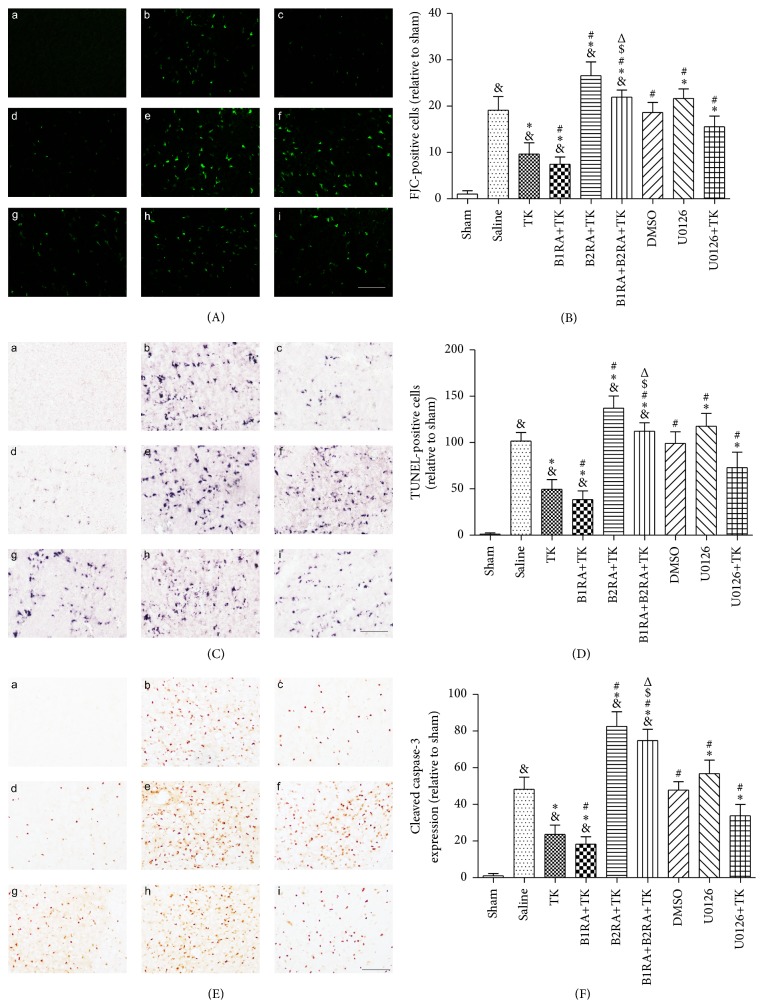
(A) FJC staining was performed 24 h after MCAO to assess neuron degeneration. (B) The number of FJC-positive cells was counted 24 h after MCAO. (C) TUNEL staining was performed 24 h after MCAO to assess apoptosis in neurons. (D) The number of TUNEL-positive cells was counted 24 h after MCAO. (E) Cleaved caspase-3 staining was performed 24 h after MCAO to assess neuron apoptosis. (F) The number of cleaved caspase-3-positive cells was counted after MCAO. (a) Sham; (b) saline; (c) TK; (d) B1RA+TK; (e) B2RA+TK; (f) B1RA+B2RA+TK; (g) DMSO; (h) U0126; (i) U0126+TK. Scale bar = 100 mm. Data are expressed as mean ± SD; *n* = 3 rats/group. Six representative microscopic fields were analyzed for each rat (^&^ *P* < 0.05 versus sham group; ^*∗*^ *P* < 0.05 versus saline group; ^#^ *P* < 0.05 versus TK group; ^$^ *P* < 0.05 versus B1RA+TK group; ^Δ^ *P* < 0.05 versus B2RA+TK group).

Neuronal degeneration was evaluated by FJC staining. Relative to the sham operation group, 24 h of reperfusion after MCAO stimulated a 19.12 ± 2.92-fold increase in FJC-positive cells in the ischemic cortex. Administration of TK decreased the relative density of FJC-positive cells compared to saline (*P* < 0.05, Figures 5(A) and 5(B)). The efficacy of TK treatment was enhanced with the B1R antagonist but reversed with the B2R antagonist (*P* < 0.05). The number of degenerated neurons in the B1RA+B2RA+TK group was significantly greater than that in the TK and B1RA+TK groups but less than that in the B2RA+TK group (*P* < 0.05). U0126 pretreatment aggravated neuronal injury (*P* < 0.05) and weakened the benefit of TK (*P* < 0.05).

TUNEL staining and immunohistochemical staining for cleaved caspase-3 were used to verify the antiapoptosis effect of TK in the ischemic cortex. The presence of apoptotic cells in each group was consistent with the extent of degenerated neurons (Figures 5(C), 5(D), 5(E), and 5(F)).

### 3.6. Activity of the ERK1/2 Signaling Pathway

**Figure 6:**
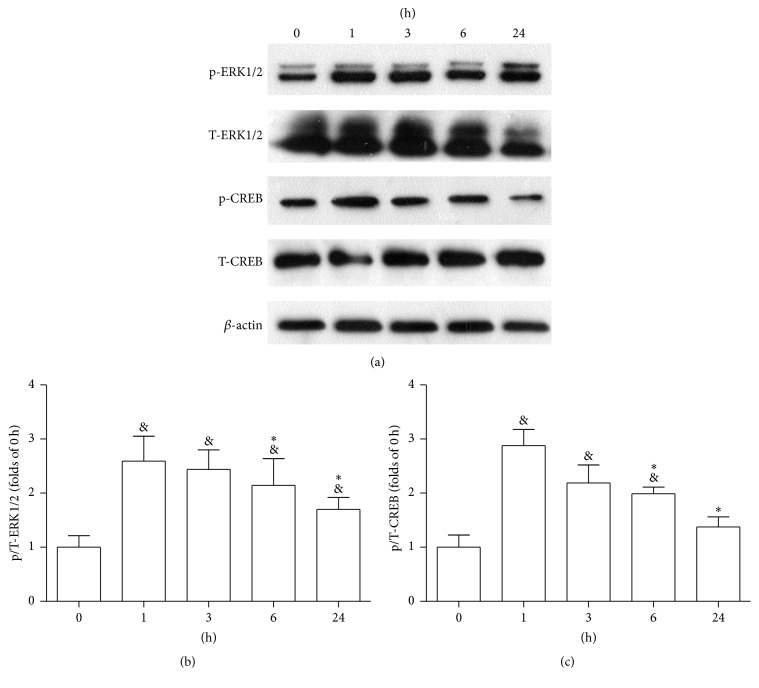
(a) Western blotting was performed to evaluate the expression levels of p-ERK1/2 and p-CREB. Brain tissues were extracted at 0 h, 1 h, 3 h, 6 h, and 24 h after MCAO. The phosphorylation of ERK1/2 (b) and CREB (c) peaked at 1 h. Data are expressed as mean ± SD; *n* = 3 rats/group (^&^ *P* < 0.05 versus 0 h; ^*∗*^ *P* < 0.05 versus 1 h).

Compared to corresponding expressions before MCAO, the relative levels of p/T-ERK1/2 and p/T-CREB showed remarkable increases with reperfusion; these levels showed fold increases of 2.6 and 2.8 after 1 h of reperfusion, respectively (*P* < 0.05, Figure 6). Thus, 1 h of reperfusion was chosen as the optimal time point for measuring the expression of signaling proteins.

**Figure 7:**
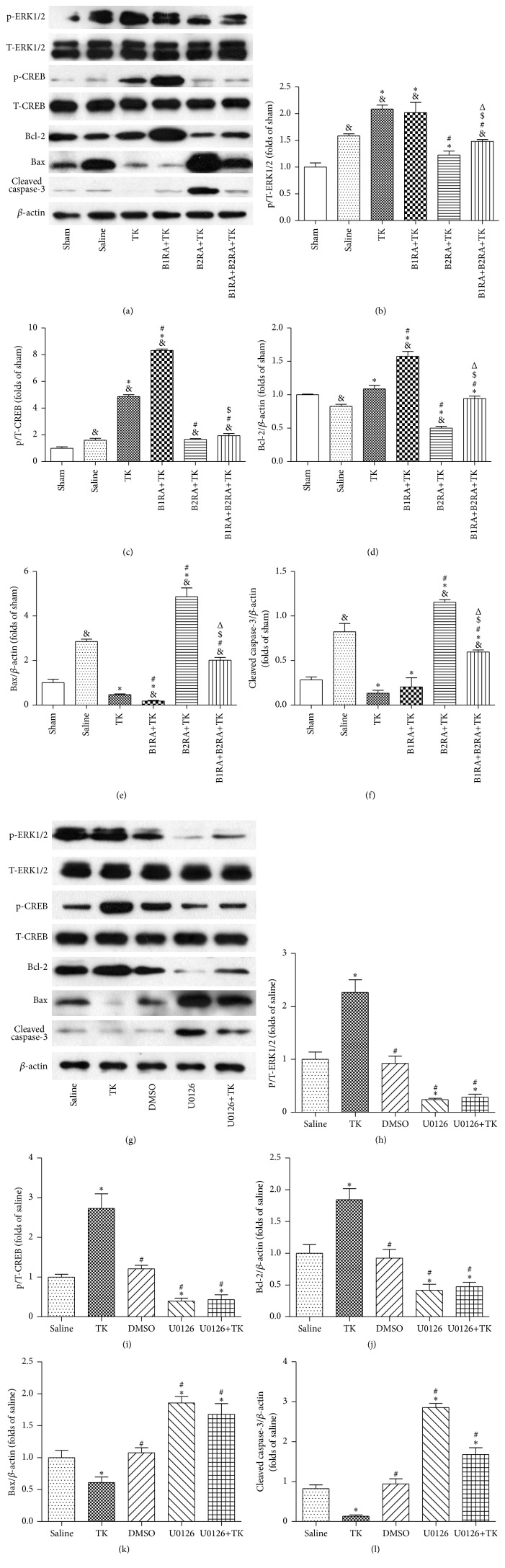
(a) Western blotting was performed to evaluate the activation levels of p-ERK1/2, p-CREB, Bcl-2, and Bax. The levels of p-ERK1/2 (b), p-CREB (c), Bcl-2 (d), Bax (e), and cleaved caspase-3 (f) are expressed as mean ± SD; *n* = 4. (g) Western blotting was performed to evaluate the activation levels of p-ERK1/2, p-CREB, Bcl-2, and Bax. The levels of p-ERK1/2 (h), p-CREB (i), Bcl-2 (j), Bax (k), and cleaved caspase-3 (l) are expressed as mean ± SD; *n* = 3 (^&^ *P* < 0.05 versus sham group; ^*∗*^ *P* < 0.05 versus saline group; ^#^ *P* < 0.05 versus TK group; ^$^ *P* < 0.05 versus B1RA+TK group; ^Δ^ *P* < 0.05 versus B2RA+TK group).

TK treatment enhanced the relative expression of p/T-ERK1/2, p/T-CREB, and Bcl-2/*β*-actin compared to the sham group at 1 h of reperfusion after MCAO, with higher values compared to the saline group (*P* < 0.05, Figure 7). Pretreatment with a B1R antagonist further elevated the levels of p/T-CREB and Bcl-2/*β*-actin in TK-treated animals (*P* < 0.05). However, the B2R antagonist abated the upregulation of p/T-ERK1/2, p/T-CREB, and Bcl-2/*β*-actin generated by TK treatment, and simultaneous pretreatment with the B1R and B2R antagonists had a similar effect (*P* < 0.05). However, the levels of all of the above proteins in the B1RA+TK group were higher than those in the B1RA+B2RA+TK group, whereas the p/T-ERK1/2 and Bcl-2/*β*-actin levels in the B2RA+TK group were significantly lower (*P* < 0.05). In contrast, the elevated levels of Bax and cleaved caspase-3 expression stimulated by I/R were downregulated by TK treatment (*P* < 0.05). The inhibitory effect of TK on Bax expression was further amplified by the B1R antagonist but was weakened by the B2R antagonist (*P* < 0.05). The suppression effect of TK on cleaved caspase-3 was not changed significantly by B1R antagonist but impaired by B2R antagonist (*P* < 0.05). Furthermore, administration of B1RA+B2RA+TK decreased the levels of Bax and cleaved caspase-3 compared to those observed in the B2RA+TK group but elevated the levels in comparison to the B1RA+TK group (*P* < 0.05).

Compared to saline treatment, injection of U0126 before MCAO resulted in decreased expression of p/T-ERK1/2, p/T-CREB, and Bcl-2/*β*-actin compared to the saline group (*P* < 0.05, Figure 7). Additionally, U0126 treatment increased the levels of Bax and cleaved caspase-3 relative to the saline group (*P* < 0.05). Moreover, pretreatment with U0126 impaired the effects of TK treatment (*P* < 0.05). During the experiments, the total immunoreactive ERK1/2 and CREB levels were rarely changed by MCAO surgery or other interventions.

## 4. Discussion

In this study, we observed the neuroprotective effects of TK on I/R injury in streptozotocin-induced diabetic rats, involving the reduction of brain edema and infarct volume, suppression of inflammation and apoptosis, and amelioration of ischemia-induced behavioral deficits. TK led to upregulated expression of p-ERK1/2, p-CREB, and Bcl-2 proteins and downregulated expression of Bax and cleaved caspase-3 proteins in the cerebral I/R injury of diabetic rats. Additionally, the neuroprotective effects of TK were promoted by a B1R antagonist but were abrogated by treatment with a B2R antagonist or ERK1/2 inhibitor.

Early inflammation contributes to brain damage following I/R. Activated microglia and neutrophils are central to the inflammatory response and can release cytokines. Furthermore, neuronal degeneration and apoptosis and inflammatory responses in ischemic areas can be aggravated by hyperglycemia [29]. Previous studies have shown that hyperglycemia enhances microglia activation and neutrophil migration, which further worsened postischemia injury [30, 31]. This study revealed that immediate TK treatment after MCAO attenuated I/R-induced neuronal degeneration and cellular apoptosis, inhibited neutrophil migration and microglia activation, and reduced the levels of proinflammatory cytokines in diabetic rats. Furthermore, there were notably improved neurological outcomes in the TK-treated rats, indicating TK's involvement in the brain I/R process.

Pharmacological B2R blockade before TK treatment could boost neuron loss, microglia activation, neutrophil migration, and the levels of proinflammatory cytokines, suggesting that the neuroprotective effects of TK were mainly mediated by B2R. Our observations are consistent with the published literature showing that TK protects neurons from ischemic injury through activation of B2R [32]. Additionally, TK was demonstrated to inhibit I/R-induced apoptosis and inflammation in the brains of nondiabetic rats, and the efficacy was blocked by B2R antagonists [33]. However, in the present study, blockade of B1R before MCAO facilitated the actions of TK, indicating that activation of B1R exaggerated cerebral I/R injury in diabetic rats. Our recent findings showed that the upregulation of B1R in diabetic rats aggravated cerebral I/R injury compared with nondiabetic rats. Therefore, the B1R pharmacological antagonist plays a neuroprotective role in acute ischemia in diabetic rats [22], and this phenomenon is consistent with a report demonstrating that B1R-knockout nondiabetic mice suffering brain I/R injury have smaller infarct volumes and less postischemic inflammation [34]. Therefore, a combination of the B1R antagonist with TK treatment may provide a novel strategy for the treatment of stroke with diabetes. Additionally, simultaneous antagonism of B1R and B2R reversed the beneficial effects of TK, suggesting that the protection induced by TK was mediated primarily by B2R, although B1R activated by TK showed a detrimental effect on brain I/R injury in diabetic rats [22]. Thus, our results demonstrated that activation of B2R in cerebral I/R damage in diabetic rats suppressed inflammation, enhanced cell survival, and improved neurological functions, whereas activation of B1R produced the opposite effects.

A previous study on myocardial ischemia demonstrated a cardioprotective effect of the B2R agonist in nondiabetic mice and the B1R agonist in diabetes via inhibition of GSK-3*β* [35]. Possible explanations for this discrepancy include differences in animal species, tissues, organs, and I/R injury models. Moreover, in our findings, inactivation of B2R before MCAO resulted in a lower level of ERK1/2 phosphorylation than TK intervention alone in the ischemic brain, which was contrary to B1R inactivation. These observations suggest that activation of B2R signaling induced by TK leads to the phosphorylation of ERK1/2 in the diabetic cerebrum, indicating that B2R may be the main upstream protein in the ERK1/2 signaling pathway activated by TK.

The ERK1/2 pathway is known to be activated by various stimuli, such as oxidative stress, ionic imbalance, activation of glutamate receptors, and growth factors [36], and this pathway also plays a role in TK-mediated protection against I/R injury in orthoglycemic conditions [17–19]. Previous studies have demonstrated that the stimulation of B2R caused NO production and NO could stimulate ERK [37, 38]. Therefore, the activation of B2R may lead to the upregulation of p-ERK1/2. Here, we also observed that the ERK1/2 pathway was one of the main signaling pathways involved in TK-mediated anti-inflammation and antiapoptosis following cerebral I/R in diabetes. TK treatment during reperfusion after MCAO in diabetic rats induced upregulation of p-ERK1/2, whereas pretreatment with the ERK1/2 pathway inhibitor U0126 before MCAO produced adverse effects that were similar to those for the B2R antagonist. U0126 was reported to significantly aggravate neurological dysfunction, increase infarct volumes and edema, and exacerbate inflammation and apoptosis [39, 40]. Our findings further showed that pretreatment with U0126 could partially but significantly counteract the efficacy of TK against cerebral I/R injury in diabetic rats. This phenomenon hints that the actions of TK against brain I/R injury in diabetic rats might involve other pathways besides the ERK1/2 signaling pathway. However, the ERK1/2 inhibitor completely suppressed the activation of p-ERK1/2, p-CREB, and Bcl-2 and promoted the activation of Bax and cleaved caspase-3 in TK-treated group, indicating that ERK1/2 may be the main upstream protein in this signaling pathway activated by TK.

CREB, as a transcription factor, participates in synaptic plasticity, memory. and survival. Phosphorylation of CREB by p-ERK1/2 not only displays neuroprotection in stroke animals [36, 41] but also prevents postischemic inflammation and neuronal damage [42]. Our data showed that the level of phosphorylated CREB changed with the expression of p-ERK1/2. Phosphorylated CREB was reported to stimulate expression of the antiapoptotic protein Bcl-2 and the reduction of caspase-3, which are associated with neuronal survival [43–46]. Bax and Bcl-2 are members of the Bcl-2 protein family and are associated with neuronal apoptosis. Our study revealed that TK could stimulate the signal pathway of ERK1/2-CREB-Bcl-2 and suppress the Bax and cleaved caspase-3 expressions. Additionally, pretreatment with the B1R antagonist strengthened the actions of TK on the abovementioned signaling pathways, whereas the B2R antagonist or ERK1/2 inhibitor U0126 produced the opposite effects on these signaling proteins. We also noticed sharp increases in degenerative and apoptotic neurons as well as inflammatory reactions after inhibition of the B2R or ERK1/2 pathway. The present findings were consistent with a previous study in which U0126 was shown to counteract the neuroprotection of ERK-CREB in transient global ischemia [47]. It is likely that the anti-inflammatory activity induced by TK through B2R involves enhanced phosphorylation of ERK1/2 and subsequently increases the phosphorylation of CREB, which in turn suppresses proinflammatory cytokine secretion in the damaged tissue and reduces microglia and neutrophil migration. According to our observations, TK might produce antiapoptotic effects through B2R and activation of the ERK1/2-CREB pathway, in addition to promoting Bcl-2 formation, suppressing Bax and cleaved caspase-3 expressions, and reducing cell degeneration and apoptosis.

Various drugs for stroke were neuroprotective preclinically but proved unsuccessful in clinical trials subsequently. Using healthy animals as the model to evaluate the efficacy of candidate drugs may be one of the most common problems. Recommendations issued by Stroke Therapy Academia Industry Roundtable (STAIR) Committee showed that drug intended for clinical trials should be administrated to animals accompanied with hypertension, diabetes, and hypercholesterolemia if it was demonstrated to be effective in healthy animals [46]. TK was demonstrated to offer neuroprotection by previous work in orthoglycemic models; however, the research on the role of TK in stroke animals with diabetes remains scarce. Therefore, it is meaningful to prove that TK could ameliorate the prognosis of I/R injury in hyperglycemic conditions.

In conclusion, early administration of TK could provide robust resistance to I/R damage in the diabetic brain. This neuroprotective effect was attributed, in the present study, to the antiapoptotic and anti-inflammatory effects mediated primarily through B2R and the ERK1/2-CREB-Bcl-2 pathway. Thus, our findings support the use of TK as a therapeutic approach to reduce the effects of brain I/R insults in diabetic subjects.
